# Depression and anxiety disorders in patients with atrial fibrillation undergoing a pulmonary vein isolation: A systematic literature review and meta-analysis

**DOI:** 10.1038/s41598-026-42473-4

**Published:** 2026-03-12

**Authors:** Sebastian Weyand, Peter Seizer, Florian Junne, Caroline Rometsch

**Affiliations:** 1 Medizinische Klinik II-Kardiologie Und Angiologie, Ostalb-Klinikum Aalen, Im Kälblesrain 1, 73430 Aalen, Germany; 2 University of Ulm, 89069 Ulm, Germany; 3 Department of Psychosomatic Medicine and Psychotherapy, University medicine Magdeburg, Leipzigerstr. 44, Magdeburg, Germany 39120; 4 Department of Psychosomatic Medicine and Psychotherapy, University of Tübingen, Osianderstr. 5, 72076 Tübingen, Deutschland

**Keywords:** Depression, Anxiety, Pulmonary vein isolation, Ablation, Prevalence, Psychocardiology, Epidemiology, Psychology, Cardiology

## Abstract

Atrial fibrillation (AF) is globally the most prevalent arrhythmia for which pulmonary vein isolation (PVI) is an effective intervention. A high comorbidity with mental disorders, in particular depressive and anxiety disorders, is described; however, a systematic synthesis of the existing literature is missing. This systematic review and meta-analysis aim to fill in this gap and to examine the prevalence of depressive and anxiety disorders with in-depth analyses of associated factors and predictors of recurrence of AF. A systematic search in PubMed, Web of Science, Trials, Embase, PsycInfo, and the Cochrane library was conducted. Articles on depressive and anxiety disorders according to the International Classification of Diseases (ICD) or the Diagnostic and Statistical Manual of Mental Disorders (DSM) in English-language in adults with AF undergoing PVI were included. Preferred Reporting Items for Systematic reviews and Meta-Analyses (PRISMA) guidelines were followed. Prevalence rates were synthesized, and meta-analyses were conducted to calculate the pooled point prevalence of depressive and anxiety disorders applying generalized linear mixed-effects models. Subgroup analyses on the prevalence including the age, sex, AF type, comorbid cardiovascular; meta-correlations and meta-regressions were performed. Predictors of recurrence of AF were calculated using logistic regressions. Eighteen studies with a total of 9,691 observations for depressive disorders and 8,855 observations for anxiety disorders were included. The meta-analyses revealed an overall point prevalence of 20.10% [95% Confidence interval (CI): 13.31% to 29.18%] for depressive disorders and 25.18% [95% CI: 11.71% to 46.08%] for anxiety disorders in patients undergoing PVI. Subgroup analyses indicated higher prevalence rates of depressive disorders in younger patients, those with paroxysmal AF, and patients with comorbid conditions such as heart failure, diabetes, hypertension. Older patients and those with cardiovascular disorders showed a higher prevalence of anxiety disorder. The correlation between pre- and post-PVI mental health status was strong (*r* = .99 for depression), but neither pre-ablation depression (OR = 1.022, *p* = .638) nor anxiety (OR = 1.003, *p* = 0.830) significantly predicted AF recurrence. Meta-regression analyses did not identify significant moderators explaining existing between-study heterogeneity. Depressive and anxiety disorders are highly prevalent among patients with AF undergoing PVI, particularly with higher rates of depression in younger individuals, those with paroxysmal AF, and patients with comorbid cardiovascular conditions. While mental health status improved post-PVI, neither pre-ablation depression nor anxiety were significant predictors of AF recurrence. This suggests that mental health diagnostics and interventions should be integrated into routine care, although they may not directly impact AF recurrence after PVI. Current guidelines address the management of the psychosocial dimension and comorbid mental disorders of AF insufficiently, indicating an unmet need for structured psychosomatic co-management within an interdisciplinary, patient-centered care framework. Following a biopsychosocial approach may be beneficial and warrants further targeted investigation in AF populations.

**Supplementary Information:**

The online version contains supplementary material available at 10.1038/s41598-026-42473-4.

## Introduction

Atrial fibrillation (AF) is the most prevalent arrhythmia worldwide, with an estimated point prevalence of 1–2% in the adult population^1^. The prevalence rises as high as 17% in patients aged over 80^2^. This condition leads to significant economic and public health challenges, including an increased likelihood of stroke, higher hospitalization rates, and reduced quality of life^3^. 

There is a high comorbidity of mental disorders in patients with AF, particularly depressive and anxiety disorders^4^. This is in line with comprehensive epidemiological data indicating overall prevalence rates of 31.3% for depression and 32.9% for anxiety among patients with any cardiac disorder^5^. Comorbid mental disorders contribute to decreased quality of life, increased health care utilization, and ultimately, elevated morbidity rates among patients with AF^6^, with patients suffering from more severe depressed mood in persistent AF compared to paroxysmal AF, even when controlling for similar symptom burden and relevant factors^7^. Depressive disorders are one of the leading causes of disability worldwide^8^, characterized by the presence of a sad, empty, or irritable mood, accompanied by somatic and cognitive changes that significantly impair functioning. Further symptoms such as fatigue, reduced motivation, and sleep disruptions are common in patients with depressive episodes^9^. Anxiety disorders are characterized by persistent and excessive worry, fear, or anxiety about various aspects of daily life, which can be accompanied by unspecific somatic symptoms such as dyspnoea, thoracic pain, dizziness^10^.

AF can precipitate the onset of depressive and anxiety disorders among patients. Conversely, individuals with depressive or anxiety disorders may have an increased risk of developing AF^11^ resulting in a clear association between AF symptom severity and both depressive and anxiety disorders^12^. Over time, the increasing prevalence of depressive disorders affects the progression of AF^13^, similarly, anxiety disorders can influence the trajectory of AF^14^, often due to skipped medical appointments and less healthy lifestyle choices^15^.

Acknowledging the complex interplay between mental health disorders and AF underscores the need for holistic treatment strategies. Early rhythm-control therapy is associated with a lower risk of cardiovascular outcomes, such as death from cardiovascular causes or stroke^16^ and can be addressed with both pharmacological and non-pharmacological interventions^17^. Pulmonary vein isolation (PVI), a non-pharmacological intervention, has been recommended by the European Society of Cardiology^18^ due to its proven effectiveness in reducing recurrent AF and enhancing patients’ quality of life^19^. PVI involves the electrical separation of the pulmonary veins from the left atrium by performing circular ablations around the pulmonary vein ostia to isolate trigger foci^20^.

However, in patients undergoing PVI, the evidence base remains fragmented and difficult to translate into practice because studies vary widely in populations (e.g., age, AF subtype, comorbidities), timing of assessment (e.g., pre- vs post-PVI), measurement approaches, and outcome definitions (e.g., recurrence monitoring). Although previous reviews examined depression^21^ or anxiety^14^ as predictors of AF recurrence after catheter ablation, a comprehensive synthesis that (i) quantifies pooled prevalence of depressive and anxiety disorders in patients undergoing PVI, (ii) evaluates pre- vs post-PVI mental health status, and (iii) examines associated demographic and clinical factors across AF subtypes is still lacking. A focused synthesis in PVI cohorts is therefore needed to inform clinical screening strategies and interdisciplinary care pathways.

This study aims to systematically review the literature on depressive and anxiety disorders in patients with AF undergoing PVI. Additionally, it seeks to synthesize and meta-analyze the prevalence rate of these mental health conditions, examining both pre- and post-PVI phases, while exploring associated factors and predictors of AF recurrence through subgroup analyses and meta-regression techniques.

## Methods

### Eligibility criteria

English articles published in peer-reviewed journals on patients aged 18 and over with AF undergoing PVI and comorbid depressive and/or anxiety disorders were included. Articles with observational (e.g. cross-sectional studies, cohort studies, longitudinal studies) or experimental study designs (e.g. randomized controlled trials) had to present the point prevalence of depressive and/or anxiety disorders in patients with AF undergoing PVI. Depressive or anxiety disorders had to be diagnosed according to the International Classification of Diseases (ICD) or the Diagnostic and Statistical Manual of Mental Disorders (DSM) (in any revision) using standardized and validated psychometric instruments (e.g., Self-Rating Depression Scale (SDS), Beck’s Depression Inventory (BDI), Hospital Anxiety and Depression Scale (HADS), Major Depression Inventory (MDI), Center for Epidemiologic Studies Depression Scale (CESD), State-Trait Anxiety Inventory (STAI), WHO-5 Well-Being Index, and Zung Self-Rating Anxiety Scale (SAS)). Studies were excluded if they referred to schizophrenia, special populations (e.g., veterans), book chapters, or case reports.

### Information sources and search strategy

A comprehensive search was conducted from inception until September, 15^th^ 2024 across various databases, including PubMed, Web of Science, Trials, Embase, PsycInfo, and the Cochrane library. Additionally, grey literature (i.e., Open Grey) was included. The search terms combined terms related to ablation of AF with those referring to mental disorders, using the Boolean operator ‘AND’ (see online supplementary material). The full search strategy for PubMed is presented in Table S1 (online supplementary material). To streamline the review process, the open-source tool Rayyan^22^ was used to remove duplicates. This systematic review was conducted in accordance with the Preferred Reporting Items for Systematic reviews and Meta-Analyses (PRISMA) guidelines^23^. In addition, the methodological approach followed key principles from the Cochrane Handbook for Systematic Reviews of Interventions, particularly regarding literature search, study selection, and meta-analytic procedures^24^. Article screening and full-text review were independently performed by two reviewers (CR and SW), with any disagreements resolved through discussion. The study protocol was pre-registered with PROSPERO (registration number: CRD42023440497)^25^.

### Data extraction and quality assessment

Data referring to publication details, participant demographics, diagnosis of depressive and/or anxiety disorder, types of AF, recurrence, ablation details, comorbidities, medications, assessment timings, and assessment instruments were collected using a standardized form. After extraction, data were organized into themes such as the prevalence of depressive and anxiety disorders among patients with AF, before and after the procedure, the impact of PVI on these mental health disorders, the impact of depressive and anxiety disorders on the recurrence of AF after PVI and predictors of patient improvement, hospital readmission, and outcomes post-PVI (Table S2, online supplementary material). The methodological quality of the studies was independently assessed by reviewers CR and SW using the Joanna Briggs Institute’s Critical Appraisal Checklist for Studies Reporting Prevalence Data (JBI)^26^. This checklist includes nine criteria that evaluate crucial aspects such as the appropriateness of the sample frame, sampling method, sample size adequacy, definition of study subjects and setting, validity of measurement tools, outcome measurement consistency, appropriateness of statistical analyses, statistical precision, and response rates. Each criterion is rated on a 4-point Likert scale (yes, no, unclear, not applicable), with a total possible score of 9, and the results are reported in the online supplementary material.

### Statistical analysis

Data were analyzed using R Studio software (version 4.3.0), employing the *metaprop* function from the meta package (version 6.2.1). Prevalence rates for depressive and anxiety disorders were derived from the original studies. A generalized linear mixed-effects model (GLMM) was applied to calculate an overall point prevalence rate for both conditions, applying a logit transformation to the proportions. Between-study heterogeneity was examined by estimating *τ*^2^ using a Maximum-likelihood estimator, in addition to assessing I^2^-, Q-statistics, and prediction intervals. The results are presented with 95% CI, based on a Clopper-Pearson distribution, and illustrated with forest plots. Subsequent subgroup analyses aimed to evaluate the overall point prevalence regarding the depression severity (moderate versus severe) and the AF recurrence. Pre- and post-intervention calculations were conducted for the SDS scale. In contrast, for the HADS scale, only pre-intervention data were analyzed due to missing post-intervention data. To quantify the severity of depressive disorders in patients prior and post-PVI, a restricted maximum-likelihood estimator for tau-squared and the Hartung-Knapp adjustment for random effects was used. Further subgroup analyses were conducted to examine the impact of various clinical and demographic factors on the prevalence of depression and anxiety disorders including the type of AF (paroxysmal vs. persistent), presence of comorbid heart failure, sex (proportion of female patients in the sample), age group, presence of comorbid diabetes mellitus, and presence of comorbid arterial hypertension. Subgroup analyses were performed applying a random-effects model to account for potential heterogeneity between studies. The effect sizes were reported as pooled proportions with 95% CI. To evaluate the relationship between pre- and post-ablation mental health status and the impact of pre-ablation depression on AF recurrence, correlation and logistic regression analyses were conducted. Correlation coefficients were calculated to assess the association between pre- and post-ablation depression and anxiety scores. Additionally, logistic regression was employed to determine whether pre-ablation depression and anxiety were significant predictors of AF recurrence employing a random intercept logistic regression model with a maximum-likelihood estimator for tau^2^, logit transformation, and Clopper-Pearson confidence intervals. To explore potential sources of between-study heterogeneity, meta-regression analyses were conducted using the *metareg* function. Analyses were based on logit-transformed proportions using restricted maximum likelihood (REML) estimation. Numerical moderators were z-standardised to allow interpretation of regression coefficients as odds ratios (OR) per one standard deviation (SD) increase in the moderator. Categorical moderators were dummy-coded, with predicted proportions reported for each category. For AF recurrence, the examined moderators included follow-up duration (months), mean age, sex (proportion of female participants), AF type (paroxysmal vs. persistent), ablation technique, and baseline prevalence of depression. For the prevalence of anxiety and depressive disorders, moderators included mean age, sex (proportion of female participants), study region (Europe vs. non-Europe). Statistical significance was assessed using Knapp–Hartung adjusted t-tests. The Egger’s test was applied to test for publication bias, and a funnel plot was produced (Figure S1 and S2, online supplementary material). Risk of bias analyses were conducted applying a meta-regression to assess the quality of the studies^27^.

## Results

The literature search resulted in a total of 2,735 articles, from which 97 were selected for full-text screening. Finally, 18 articles met the inclusion criteria and were categorized according to the following themes: 1) the prevalence of depressive and anxiety disorders among patients with AF, before and after the PVI procedure (*n* = 14); 2) the impact of PVI on these mental health disorders (*n* = 1); 3) the impact of depressive and anxiety disorders on the recurrence of AF after PVI (*n* = 2); and 4) predictors of patient improvement, hospital readmission, and outcomes post-PVI (*n* = 1).

## Results of the systematic review

### Study description

#### Study demographics and characteristics of the study participants

Of the included 18 articles, eight were conducted in Europe^6,28–34^, six in Asia^35–40^, three in Australia^41–43^, and one the USA^44^. The publication years spanned from 2012^30,40^ to 2024^43^. Sample sizes varied from 20^42^ to 5,110 participants^6^ with mean ages ranging from 55^40^ to 67 years^39^. The proportion of females in the study cohorts ranged from 19%^38^ to 50%^40^. The most commonly reported somatic comorbidities were hypertension, diabetes mellitus, heart failure, prior stroke, coronary artery disease. Hypertension prevalence ranged from 21.6%^38^ to 78.8%^44^ of patients, diabetes mellitus from 2.5%^38^ to 23.9%^44^, heart failure from 1.5%^30^ to 47%^43^, prior stroke from 5%^42^ to 14%^36^, and coronary artery disease from 9.75%^38^ to 23%^31^.

Five studies reported that patients suffered from paroxysmal AF^29,35,37,40,44^ while one study reported on persistent AF^43^ and ten studies reported participants with both paroxysmal and persistent AF^28,30–32,34,36,38,39,41,42^ with the proportion of paroxysmal AF ranging from 37%^28^ to 72.9%^38^.

#### Study designs

The included studies primarily used prospective observational designs, with two comparing PVI directly to antiarrhythmic drug (AAD) therapy^37,40^. Other study designs included a cross-sectional retrospective analysis^44^, a cross-sectional survey^33^, a retrospective cohort study^34^, a retrospective registry-based cohort study^6^, a randomized clinical trial^41^, and a prespecified subanalysis of a randomized clinical trial^43^. Intervention groups were patients receiving PVI, with numbers ranging from 20^42^ to 1,293^44^. Four studies included control groups of patients on AAD therapy, allowing for comparison between treatments^37,40–42^. For a detailed overview of the studies and their respective designs, please refer to Table 1.

**Table 1:** Study design of the included studies with description of the patients’ characteristics and number of the intervention and, if applicable, control group.

Authors	Year	Study design	Intervention group	Control group
Al-Kaisey et al.^41^	2023	randomized clinical trial	Patients with AF undergoing PVI (n = 52)	Patients with AF receiving antiarrhythmic drug therapy (n = 48)
Charitakis et al.^28^	2017	prospective observational study	Patients with AF undergoing PVI (n = 192)	Not applicable
Du et al.^39^	2023	prospective observational study	Patients with AF undergoing PVI (n = 428)	Not applicable
Efremidis et al.^29^	2014	prospective observational study	Patients with paroxysmal AF undergoing PVI (n = 57)	Not applicable
Fichtner et al.^30^	2012	prospective observational study	Patients with AF undergoing PVI (n = 133)	Not applicable
Hasebe et al.^35^	2020	prospective observational study	Patients with paroxysmal AF undergoing PVI (n = 35)	Not applicable
Hobensack et al.^44^	2023	cross-sectional retrospective analysis	Patients with AF undergoing PVI (n = 1293)	Not applicable
Jeon et al.^38^	2017	prospective observational study	Patients with AF undergoing PVI (n = 236)	Not applicable
Jia et al.^36^	2019	prospective observational study	Patients with AF undergoing PVI (n = 448)	Not applicable
Pavlicek et al.^31^	2022	prospective observational study	Patients with AF undergoing PVI (n = 118)	Not applicable
Raileanu et al.^34^	2023	retrospective cohort study	Patients with AF undergoing PVI (n = 295)	Not applicable
Risom et al.^32^	2019	nationwide cross-sectional survey	Adults post-ablation for AF or Atrial Flutter (n = 462)	Not applicable
Risom et al.^33^	2023	cross-sectional survey	Patients with AF undergoing PVI (n = 929)	Not applicable
Sang et al.^37^	2013	prospective, non-randomized comparative study	Patients with paroxysmal AF undergoing PVI (n = 82)	Patients with paroxysmal AF receiving antiarrhythmic drug therapy (n = 84)
Segan et al.^43^	2024	prespecified subanalysis	Patients with AF undergoing PVI (n = 338)	Not applicable
Teppo et al.^6^	2022	retrospective registry-based cohort study	Patients with AF and mental health conditions (n = 2,392.22)	Not applicable
Walters et al.^42^	2018	prospective observational study	Patients with AF undergoing PVI (n = 20)	Patients with AF receiving antiarrhythmic drug therapy (n = 58)
Yu et al.^40^	2012	prospective, non-randomized comparative study	Patients with paroxysmal AF undergoing PVI (n = 97)	Patients with paroxysmal AF receiving antiarrhythmic drug therapy (n = 102)

*Note:* AF = Atrial Fibrillation, PVI = Pulmonary Vein Isolation.

#### Treatment modalities and pharmacological management of AF

Among the 15 studies reporting the type of energy for ablation, radiofrequency was exclusively used in 13 studies, in one study cryoablation was used^34^ and in one study both radiofrequency and cryoablation were used^31^. Redo-PVIs were performed in 39% of cases in one single study^30^. Follow-up assessments ranged from three months^35^ to 48 months^30^. Reported recurrence rate of AF ranged from 10.7%^36^ to 59%^38^.

Beta blockers were the most prescribed antiarrhythmic drugs (AAD), with prescriptions ranging from 21.1%^29^ to 88%^31^ of patients. Seven studies described the prescription of amiodarone^28,31,34,37,40,41,44^, while eight studies described the prescription of other AADs such as dronedarone or class I antiarrhythmics^28,29,31,34,35,37,41,42^.

### Mental disorders in patients with atrial fibrillation

Patients with AF reported to suffer from depressive disorders^42^ and anxiety disorders^44^. The prevalence of depressive disorders varied from 4.2%^6^ to 74%^36^ with younger patients showing higher prevalence rates than older patients^11^ and the prevalence of anxiety disorder ranged from 1.6%^6^ to 62.7%^31^. Segan et al. reported higher prevalence rates of both depressive and anxiety disorders in females compared to males^43^, while Risom et al. (2019) reported higher prevalence rates only for anxiety disorders^32^. Hobensack et al. (2023) found that female patients were significantly more likely to present with anxiety-related symptoms and with a broad range of other symptoms^44^. Teppo et al. (2022) reported higher prevalences of mental health disorders such as depression and anxiety in females with newly diagnosed AF^6^. However, these findings were not confirmed in Du et al. (2023)^39^ or Charitakis et al. (2017), where no significant sex differences were found for either depression nor anxiety^28^.

#### Impact of depressive and anxiety disorder on AF and recurrence

Seven studies explored the relationship and predictors of depressive and anxiety disorders in patients with AF, including their impact on the recurrence of AF and response to treatments. Depressive and anxiety disorders were found to elevate the risk of recurrent persistent AF following PVI^40^ and were associated with hospital readmissions for AF^32^. In one study the recurrence of atrial tachyarrhythmia was significantly higher in patients with a poorer mental health status and high psychological distress^36^ compared to those with stable mental health. Depressive and anxiety disorders were identified as predictors for the recurrence of AF following PVI^29^ and as predictors of arrhythmia-related symptoms such as palpitations, breathlessness during activity, tiredness, and worry/anxiety^28^. However, there was no significant difference found in the frequency of AF recurrence between patients with high and low levels of depression and anxiety^34^. Furthermore, depressive and anxiety disorders were associated with a reduced likelihood of receiving an AAD^6^.

#### Impact of PVI on depression and anxiety disorder

In 13 articles, the association between catheter ablation, including PVI, and its impact on depressive and anxiety disorders were examined. Catheter ablation, in particular PVI, demonstrated superior efficacy in reducing symptoms of these disorders^29–31,33,34,38,40,42,43^ when compared to AAD therapy^37,41^. This association was identified as being evident over the long term, with follow-ups between 6 to 12 months^33,41,43^. The improvement of depressive symptoms was more significant in patients who had successful ablations compared to those with unsuccessful ones^30^. A pre-existing depression before their ablation procedure did not significantly affect their chances of developing depression after the procedure^39^.

Reduced anxiety after PVI was found to be correlated to an increased parasympathetic reactivity to stress, as indicated by improved heart rate variability indices, suggesting a role for cardiac autonomic modification in psychological improvement^35^.

One study found that post-procedure depression is common in patients with AF after RF ablation^39^.

### Assessment tools of depression and anxiety

#### Assessment tools of depressive disorders

The psychometric assessment tools used for assessing depressive disorders included the HADS in nine studies^28,31–34,39,41–43^, the SDS in three studies^35,37,40^, the BDI in two studies^29,41^, the MDI^30^, the MHI-5^36^ and the CESD^38^ were each used in one study.

#### Assessment tools of anxiety disorder

For assessing anxiety disorders, the following psychometric assessment tools were applied: The HADS in eight studies^28,31–34,39,41,43^, the STAI in three studies^29,35,38^, the SAS in two studies^37,40^ and the WHO-5 in one study^30^. For a detailed overview of the assessment tools used to measure depression and anxiety in patients with AF, please refer to Table 2.

**Table 2:** Assessment tools for depressive and anxiety disorders among patients with atrial fibrillation.

	Results (min and max) before PVI [SD]	Results (min and max) [SD] after PVI	Authors
Assessment instrument for Depression			
HADS	3.5 [3.5]—8.2 [3.9]	3.1 [2.6]—5.8 [3.7]	^28,31–34,39,41–43^
SDS	40.3 [8.6]—49.4 [10.6]	32.0 [7.4]—38.7 [8.6]	^35,37,40^
BDI	10.2 [7.6]	5.1 [4.8]	^29,41^,
MDI	13.6 [8.6]		^30^
CESD	13.4 [9.8]		^38^
MHI-5			^36^
Assessment instrument for anxiety			
HADS	4.4 [3.6]—10.4 [4.1]	3.8 [3.8]—7.2 [3.9]	^28,31–34,39,41^
SAS	35.7 [35.7]—48.6 [11.1]	30.1 [6.1]	^37,40^
STAI	36.9 [11.4]—45.9 [9.0]	25.6 [9.1]—39.0 [7.2]	^29,35,38^
WHO-5	49.5 [24.1]	65.7 [24.0]	^30^

*Note.* HADS = Hospital Anxiety and Depression Scale, SDS = Self-Rating Depression Scale, BDI = Beck Depression Inventory, MDI = Major Depression Inventory, CESD = Center for Epidemiologic Studies Depression Scale, MHI-5 = Mental Health Inventory-5, STAI = State-Trait Anxiety Inventory, SAS = Zung Self-Rating Anxiety Scale, WHO-5 = WHO-5 Well-Being Index.

### Results of the meta-analyses

#### Prevalence of depressive disorder in patients with AF undergoing PVI

**Fig. 1:**
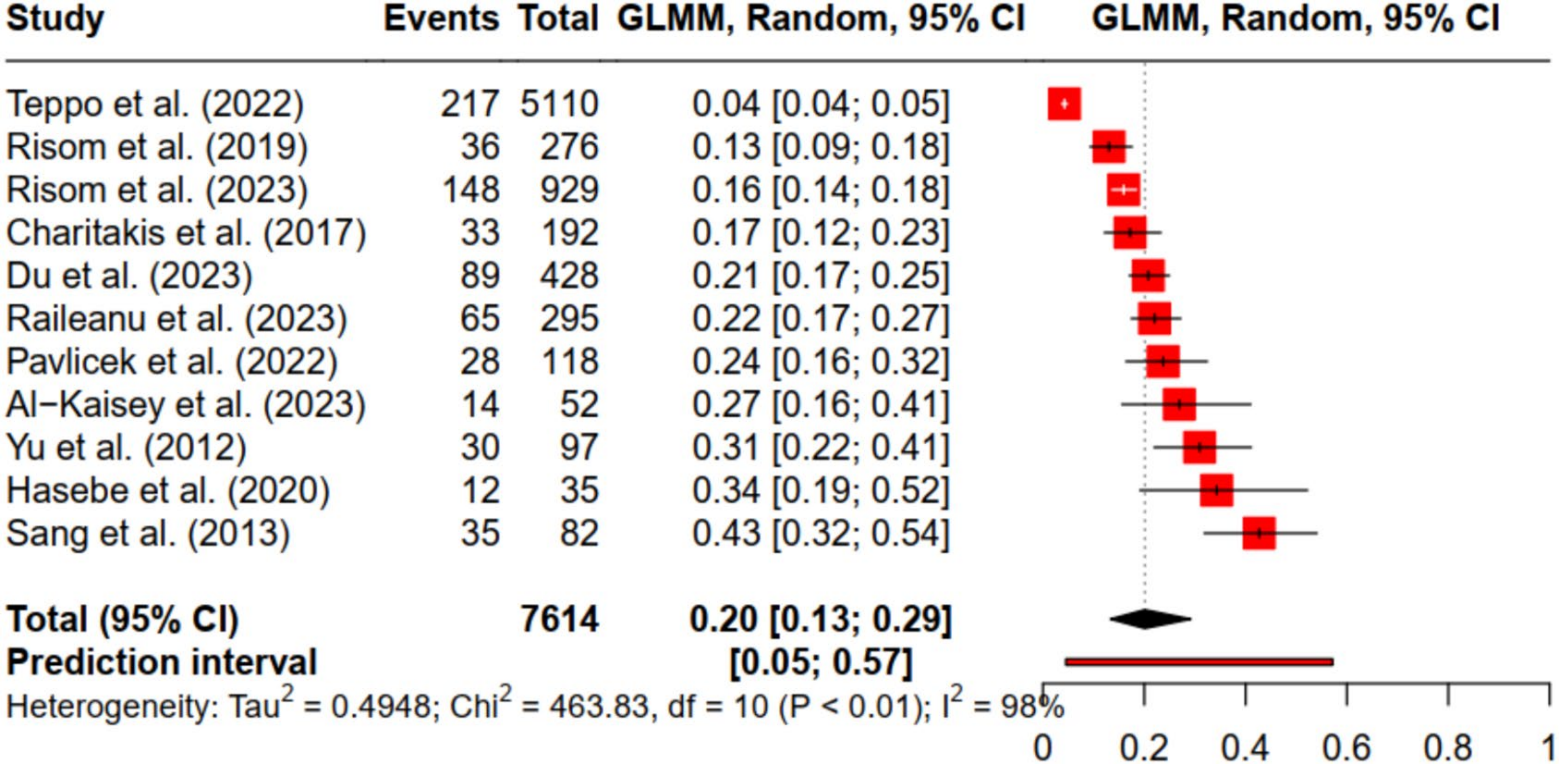
Forest plot of the prevalence of depressive disorder in patients with atrial fibrillation undergoing pulmonary vein isolation.

The overall point prevalence of depressive disorder in patients undergoing PVI, based on 9,691 observations, was 20.10% [95% Confidence interval (CI): from 13.31% to 29.18%]. The analysis showed a high degree of heterogeneity (I^2^ = 97.1%, τ^2^ = 0.495), with a prediction interval ranging from 1 to 57% (see Fig. 1). The sensitivity analysis including Jia et al. (2019) resulted in an overall prevalence of 23.86% [95%CI from 14.41% to 36.82%]. When data on the severity of depressive disorder were included, the overall point prevalence for moderate depression was 14.13% [95% CI from 3.70% to 41.30%, I^2^ = 82.8%, τ^2^ = 0.231]. For severe depression, the overall prevalence was 4.17% [95% CI: 0.13% to 12.60%, I^2^ = 89.2%, τ^2^ = 0.737].

#### Depression severity before PVI

Three studies applied the SDS resulting in a total of 214 observations. The mean depression scores from these studies ranged from 42.05 to 44.33. The random-effects model showed a slightly higher mean score of 43.71% [95% CI: 31.40 to 56.02, I^2^ = 94.7%, τ^2^ = 23.12], suggesting a moderate level of depressive symptoms among the study participants (scores range from 20 to 80). Two studies applying the HADS included a total of 586 observations, revealing mean depression scores ranging from 4.5 to 5.32. The random-effects model indicated a mean score of 5.38 [95% CI: -8.60 to 19.35, I^2^ = 95.5%, τ^2^ = 2.31]. This mean score suggests a level of depression categorized as mild, which contrasts with the categorization suggested by the SDS.

#### Depression severity in patients after PVI

The meta-analysis assessing the severity of depression using the SDS scale included two studies with a total of 214 observations. The random-effects model indicated a mean score of 35.24 [95% CI: -7.17 to 77.66, I^2^ = 94.0%, τ^2^ = 20.97]. Although this estimate is broad and includes substantial uncertainty, the mean score still suggests a moderate level of depression.

#### Prevalence of anxiety disorder in patients with AF undergoing PVI

**Fig. 2:**
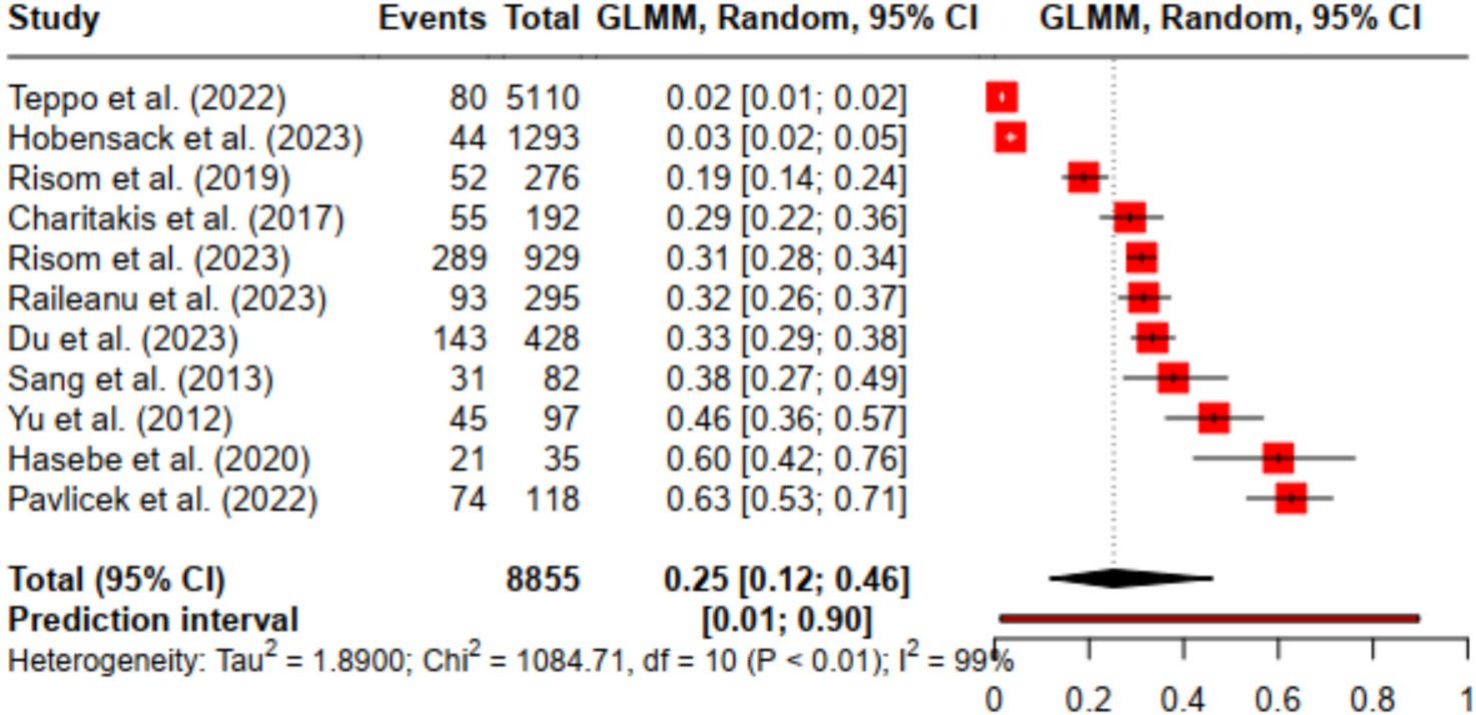
Forest plot of the prevalence of anxiety disorder in patients with atrial fibrillation undergoing pulmonary vein isolation.

For anxiety, the analysis of 8,855 observations revealed an overall point prevalence of 25.18% [95% CI: 11.71% to 46.08%]. The heterogeneity was high with an I^2^ of 99.1% and τ^2^ of 1.890. The prediction interval ranged from 1 to 90% (see Fig. 2).

#### Subgroup analysis of depression disorder

The subgroup analyses revealed significant differences in the prevalence of depression among various patient groups undergoing PVI. The prevalence of depression in relation to sex showed consistency across studies with a low proportion of female patients, resulting in an overall rate of 16.81% [95% CI: 5.12% to 43.10%]. Regarding age, younger patients exhibited a higher prevalence of depression at 35.68% [95% CI: 2.30% to 92.88%] compared to older patients at 11.16% [95% CI: 1.54% to 50.13%], with a statistically significant difference (*p* = .003). Among patients with comorbid arterial hypertension, depression rates varied significantly, ranging from 1.18% [95% CI: 0.44% to 3.11%] to 42.7% [95% CI: 32.47% to 53.56%], with a significant difference observed between subgroups (*p* < .001). Specifically, the prevalence rates for depression were higher in some studies, such as Sang et al. (2013), which reported a rate of 42.7%, compared to others like Charitakis et al. (2017), which reported a lower rate of 17.2%. Heterogeneity between studies was significant (I^2^ = 91.9%), indicating substantial variability in depression rates among patients with comorbid arterial hypertension. In patients with comorbid diabetes mellitus, the prevalence of depression ranged from 1.18% [95% CI: 0.44% to 3.11%] to 42.7% [95% CI: 32.47% to 53.56%], with significant differences observed across subgroups (*p* < .001). The highest prevalence was reported by Sang et al. (2013) with 42.7%, while the lowest was observed in Segan et al. (2024) with 1.18%. Heterogeneity between the studies was substantial (I^2^ = 91.9%), reflecting variability in depression rates among patients with comorbid diabetes mellitus.

#### Subgroup analyses of anxiety disorder

The prevalence of anxiety in relation to sex showed rates varying from 28.7% [95% CI: 22.70% to 35.44%] to 62.7% [95% CI: 53.66% to 70.95%] (*p* < 0.001), with higher prevalence observed in samples with a higher proportion of female participants compared to those with a lower proportion. Regarding age, patients with a mean age of 64 years exhibited a higher prevalence of anxiety at 62.7% [95% CI: 53.66% to 70.95%] compared to those with a mean age of 55.9 years, who had a prevalence of 37.8% [95% CI: 28.01% to 48.71%] (*p* < 0.001). Patients with paroxysmal AF had an anxiety prevalence of 37.8% [95% CI: 28.01% to 48.71%], while those in the combined AF group had a prevalence of 40.1% [95% CI: 12.00% to 76.65%], with statistically significant differences between subgroups (*p* < 0.001). Among patients with comorbid heart failure, anxiety rates varied significantly, ranging from 29.6% [95% CI: 16.00% to 54.10%] to 62.7% [95% CI: 53.66% to 70.95%] (*p* < 0.001), with higher prevalence observed in samples with a higher proportion of patients with heart failure. In patients with comorbid diabetes mellitus, the prevalence of anxiety ranged from 29.6% [95% CI: 16.00% to 54.10%] to 62.7% [95% CI: 53.66% to 70.95%] (*p* < 0.001), with higher prevalence observed in samples with a higher proportion of patients with diabetes mellitus. Similarly, the presence of comorbid arterial hypertension was associated with varying rates of anxiety, ranging from 29.6% [95% CI: 16.00% to 54.10%] to 62.7% [95% CI: 53.66% to 70.95%] (*p* < 0.001), with higher prevalence observed in samples with a higher proportion of patients with arterial hypertension.

#### Recurrence of AF

The meta-analysis on the proportion of AF recurrence included 13 studies with a total of 2,727 observations. The overall proportion of AF recurrence was calculated with 30.25% [95% CI: 12.53%; 56.76%, I^2^ = 95.9%, tau^2^ = 3.208].

#### Mental health status pre- and post-PVI and the impact on AF recurrence

The correlation analysis revealed a strong positive correlation between pre-ablation and post-ablation when a depression (*r* = 0.99) was diagnosed. Logistic regression analyses indicated that pre-ablation depression (OR = 1.022, *p* = 0.638) and pre-ablation anxiety (OR = 1.003, *p* = 0.830) were no significant predictors of AF recurrence (see Tables 3 and 4).

**Table 3:** Logistic regression analysis for pre-ablation depression.

Statistic	Odds ratio	Standard error	z value	p-value
Value	1.022	0.04603	0.471	0.638

**Table 4:** Logistic regression analysis for pre-ablation anxiety.

Statistic	Odds ratio (OR)	Standard error	z value	p-value
Value	1.003	0.01424	0.215	0.830

#### Results of the moderator analyses

For AF recurrence, meta-regression analyses across four studies indicated that none of the examined moderators were significantly associated with recurrence risk. Baseline depression prevalence (OR = 1.15, 95% CI 0.82–1.61, *p* = 0.28), follow-up duration (OR = 0.93, 95% CI 0.71–1.22, *p* = 0.46), mean age (OR = 1.07, 95% CI 0.83–1.38, *p* = 0.42), proportion of female participants (OR = 1.04, 95% CI 0.79–1.38, *p* = 0.71), AF type (paroxysmal vs. persistent; OR = 1.32, 95% CI 0.57–3.06, *p* = 0.52), ablation technique (OR = 0.88, 95% CI 0.41–1.90, *p* = 0.74) showed non-significant associations. For depression prevalence, based on five studies, none of the moderators reached statistical significance. Mean age (OR = 0.94, 95% CI 0.74–1.19, *p* = 0.43) and sex (OR = 1.08, 95% CI 0.86–1.36, *p* = 0.54) showed no significant association. A trend towards higher prevalence was observed for studies conducted outside Europe compared to those conducted in Europe (OR = 2.16, 95% CI 0.94–4.94, *p* = 0.066), corresponding to predicted proportions of 36.2% and 20.8%, respectively. For anxiety prevalence, analyses across four studies likewise found no statistically significant moderator effects. Mean age (OR = 1.05, 95% CI 0.82–1.34, p = 0.62), sex (OR = 1.02, 95% CI 0.79–1.31, p = 0.86), and study region (non-Europe vs. Europe; OR = 0.92, 95% CI 0.54–1.57, p = 0.78; predicted proportions 40.2% vs. 37.8%) were not statistically significant.

## Publication bias

The Egger test for the meta-analysis on the prevalence of depressive disorders (t = 1.91, df = 10, *p* = 0.085) and for the prevalence of anxiety disorders (t = 0.29, df = 9,* p* = 0.776) did not indicate significant publication bias.

## Risk of bias

The results revealed a higher prevalence of depressive disorders in studies with a JBI score of 8 compared to those with a JBI score of 9. There was notable heterogeneity across studies (I^2^ = 97.7%). For anxiety disorders, the analysis similarly revealed substantial heterogeneity (I^2^ = 99.0%). However, no significant differences in prevalence were found between subgroups based on JBI scores (*p* = 0.995). Specifically, the random-effects model estimated the overall proportion of high-risk studies for anxiety disorders at 21.65% [95% CI: 9.72%; 41.51%], with subgroup analyses indicating proportions of 21.48% [95% CI: 5.75%; 59.39%] for JBI score 8 and 22.58% [95% CI: 6.48%; 57.10%] for JBI score 9.

## Discussion

This systematic review revealed a prevalence of depressive disorders ranging from 4.2% to 74% and a prevalence of anxiety disorders ranging from 1.6% to 62.7% among patients with AF undergoing PVI. Meta-analyses indicated an overall point prevalence of depressive disorders at 20.10% [95% CI: 13.31% to 29.18%] and anxiety disorders at 25.18% [95% CI: 11.71% to 46.08%] among patients with AF undergoing PVI. Subgroup analyses demonstrated higher prevalence rates of depression in younger patients, patients with paroxysmal AF, while higher rates of anxiety were observed in older patients. The prevalence of both depressive and anxiety disorders was elevated in patients with comorbid heart failure, diabetes, and arterial hypertension. The meta-analysis on AF recurrence estimated an overall recurrence rate of 30.25% [95% CI: 12.53%; 56.76%]. Logistic regression analyses indicated that neither pre-ablation depression (OR = 1.016,* p* = 0.644) nor anxiety (OR = 1.003, *p* = 0.842) were significant predictors of AF recurrence.

The overall prevalence of depressive disorders with 20.10% [95% CI: 13.31% to 29.18%] indicated that approximately one fifth of patients suffer from depressive disorders. In patients with permanent AF, the prevalence of depressive disorders is comparable with 20.2% of patients having high levels of depression^45^. This highlights the importance of early diagnosis and treatment of mental disorders in patients with AF. Timing therapy around the point of PVI could be particularly effective.

When considering other cardiovascular diseases, mental comorbidities have been reported with high prevalence rates. Depressive disorders were present in 21.5% of patients with heart failure^46^. In 19.8% of patients with acute myocardial infarction with a significant proportion of patients continued to be depressed in the year after discharge^47^. In a cohort study at 3 months after acute coronary syndrome, 61.7% of patients reported anxiety or depressive disorders, and those with persistent symptoms had higher mortality compared to others^48^. Depression, with a prevalence of 15.4%, and anxiety, with a prevalence of 22.6%, are highly prevalent among patients with ICD, with rates further increasing in those who have experienced shocks^49^. A study found that patients with Brugada syndrome experience higher levels of mental distress compared to the general population^50^. In stroke survivors the prevalence of depressive disorders was 34%^51^. Patients with CVD were at elevated risk of mental disorders and the other way around an occurrence of psychiatric comorbidity after CVD diagnosis was associated with an approximately 55% higher risk of subsequent death from cardiovascular causes^52^. It appears that the prevalence of depressive disorders depends on the type of cardiac disorder, with more life-threatening and somatically disabling cardiac disorders leading to higher prevalence rates. A similar pattern was reported in the DenHeart study, which found poorer patient-reported mental and physical health among patients with heart failure and valvular disease at discharge compared to patients with other cardiac diagnoses^53^. Although differences between diagnostic groups were statistically significant, they were small and did not reach the threshold for clinical relevance. Across all groups, around 20–30% of patients reported symptoms of anxiety or depression, indicating that psychological distress is prevalent among cardiac patients in general^53^.

The overall point prevalence of anxiety disorder was 25.18% [95% CI: 11.71% to 46.08%]. Again: around one fourth of all patients with AF undergoing PVI suffer from anxiety disorder. In cardiac patients with diverse conditions, anxiety seems to be much higher with prevalence rates up to 43%^54^, emphasizing that AF is not leading to life-threatening conditions in the perception of the patients.

Younger patients exhibited significantly higher levels of depression compared to older patients. This finding mirrors previous research indicating that younger adults with AF experience greater psychological distress due to the disruption of their daily lives and future expectations^55^. Younger patients may perceive AF as a more immediate threat to their lifestyle, leading to heightened depressive symptoms, as compared to older adults who may have more experience coping with chronic conditions. However, the results indicated higher prevalence rates of anxiety in older patients compared to younger ones, although the mean age in both groups was over 55 years, with older patients showing a prevalence of 62.7% [95% CI: 53.66% to 70.95%] and younger patients showing a prevalence of 37.8% [95% CI: 28.01% to 48.71%]. Higher prevalence rates of anxiety disorders were found in female samples, which is in line with previous studies suggesting higher prevalence rates in females with cardiovascular diseases^56^. This suggests that, in the context of AF and its treatment, several factors associated with AF such as age and sex, have a stronger influence on mental health outcomes and must be considered in the diagnostic and treatment procedure.

The systematic review revealed significant variations in the assessment tools used to measure depressive and anxiety disorders. The use of multiple instruments across different studies contributes to heterogeneity, complicating direct comparisons and interpretations. In the context of epidemiological studies, psychometric tools to assess mental disorders have been shown to lead to an overestimation of prevalence estimates^57^, in turn, diagnostic clinical interviews are regarded as the most effective method for diagnosing mood disorders leading to lower prevalence rates^58^. In patients with AF undergoing PVI only psychometric tools were used, likely leading to an overestimation of the true prevalence of both depressive and anxiety disorders. Thus, it is essential for future research to include clinical interviews to determine a more accurate prevalence. Moreover, to effectively compare the severity of mental conditions, consistency in the application of assessment tools across studies is necessary. The Patient Health Questionnaire (PHQ) is internationally recognized and could serve as a standardized tool for broader comparisons; however, this tool was not used by the included studies. Implementing the PHQ in diagnosing mental disorders would facilitate more reliable data comparison across various health spectrums.

PVI not only treats AF itself but also beneficially impacts the course of associated mental health disorders. Prior studies that have suggested mental health may not be a primary driver of AF recurrence, but rather a secondary factor (confounder) that warrants careful management^52^. However, literature is inconclusive, as in studies investigating cardioversion, depressive disorders were found to be significant predictor of a relapse in patients with AF after cardioversion^21^. Also, previous meta-analyses have reported significant associations between anxiety and depression disorders and AF recurrence after catheter ablation. Zhuo et al. (2020) found a twofold increased risk of AF recurrence in patients with baseline depression^59^, while Du et al. (2022) reported that baseline anxiety was associated with higher recurrence rates^60^. In contrast, this meta-analysis found no significant predictive value for either pre-ablation depression nor anxiety. Both Zhuo et al. (2020) and Du et al. (2022) included a limited number of studies (5^60^ and 7^59^ studies, respectively). These low number of studies may have facilitated the detection of significant associations, but potentially limit generalizability. In contrast, the present study was the first to systematically evaluate both anxiety and depression simultaneously across a larger body of literature, AF subtypes, psychometric assessments, and follow-up durations. Compared with earlier meta-analyses^61–63^, the present work additionally provides pooled prevalence estimates for both depression and anxiety disorder in patients undergoing PVI, integrates pre-/post-procedure mental health findings, and examines a broader range of clinically relevant correlates to better inform screening and care pathways. By integrating this wider range of studies and applying rigorous statistical methodology, this meta-analysis provides a more conservative and generalizable estimate, with moderator analyses showing no robust predictors and substantial unexplained heterogeneity. This suggests that variability in prevalence and recurrence is unlikely to be explained by the examined study-level factors alone, underlining the need for standardized assessments and larger, well-powered studies. Given the consistently high heterogeneity, additional meta-regression analyses were conducted to explore potential sources of between-study variability. None of these moderators reached statistical significance, suggesting that the heterogeneity is not explained by any single measured factor. While this warrants cautious interpretation of the pooled estimates, the consistency in the direction of effects supports the validity of the overall findings. Despite the high heterogeneity, pooling was undertaken using a random-effects model with prediction intervals, in line with established meta-analytic practice, to provide an overall quantitative estimate and formally explore sources of variability. There is a significant body of research not limited to PVI suggesting that while AF can exacerbate symptoms of depression and anxiety, these mental health conditions also have the potential to influence the onset and progression of AF. Research indicates that anxiety is a risk factor for the onset of AF, particularly over long-term periods^64^. Additionally, patients with AF and mental health disorders, such as depression and anxiety, may experience more severe AF symptoms^65^. Therefore, clinicians are responsible to adopt a comprehensive approach including comprehensive mental health evaluations and integrated care strategies that address both the psychological and cardiovascular aspects. While PVI appears to have a positive effect on mental health, structured cardiac rehabilitation following ablation has not consistently shown additional benefits. For instance, in the CopenHeartRFA trial, mental health improved in both groups, but no significant difference was found between the intervention and control groups after six months^66^.

In the 2024 ESC Guidelines for the Management of AF, depression is identified as an AF-related outcome. Additionally, patients with AF are reported to have a higher incidence of anxiety disorders. The guidelines also note that psychological distress and suicidal ideation (being one criteria of depression) are prevalent among patients with AF. However, the guidelines do not provide specific recommendations on how to manage these psychological comorbidities in patients with AF^18^. In line, the 2023 ACC/AHA/ACCP/HRS Guideline for the Diagnosis and Management of AF, does not address mental disorders^67^. The 2024 European Heart Rhythm Association/Heart Rhythm Society/Asia Pacific Heart Rhythm Society/Latin American Heart Rhythm Society expert consensus statement on catheter and surgical ablation of AF highlights that AF can have a substantial impact on mental health. According to an observational study cited in the EHRA Guidelines, more than one-third of patients referred for AF management experienced severe psychological distress^42^. However, the guidelines do not provide specific recommendations on how to manage these psychological comorbidities^68^. Furthermore, the AFNET/EHRA consensus conference^69^ acknowledges that AF ablation reduces psychological distress more than antiarrhythmic drug therapy and states that active rhythm management should be part of the default initial treatment for all suitable patients with AF.

Notably, there is no mention of the potential benefits of psychosomatic co-management, suggesting a gap in an interdisciplinary and patient-centered care approach following a bio-psycho-social approach as is it commonly applied in the psychosomatic discipline^70^. This highlights the need for further studies to better understand the impact of psychological support in AF management. It also indicates that an expansion of the current guidelines may be necessary to incorporate strategies for addressing mental health alongside physical health in the treatment of AF patients.

This study has several limitations that need to be discussed. The use of diverse assessment tools, inclusion of studies with varying designs, inclusion of studies from diverse geographical locations and healthcare settings might affect statistical analysis and therefore affecting the generalizability of the findings. Furthermore, small sample sizes in some subgroups, particularly in the recurrence meta-analyses, reduce the statistical power and reliability of the results, increasing the risk of biased conclusions. High heterogeneity was observed across pooled prevalence estimates which is common in meta-analyses of proportions, where substantial variation in populations, settings, follow-up durations, and assessment instruments is expected even after transformation and random-effects modelling^27^. Although meta-regression analyses were performed, no significant moderators were identified, and a substantial proportion of heterogeneity remained unexplained. This limits the ability to draw final conclusions and warrants cautious interpretation of the pooled estimates.

## Conclusions

### Summary of findings

Depressive and anxiety disorders are common among patients with atrial fibrillation undergoing PVI, with pooled point prevalences of 20.1% and 25.2%, respectively. Higher depression rates were observed in younger patients, those with paroxysmal AF, and individuals with cardiometabolic comorbidities, whereas anxiety was more prevalent in older and cardiovascularly burdened samples. Although pre- and post-PVI depression scores were highly correlated, neither baseline depression nor anxiety significantly predicted AF recurrence. Meta-regression analyses did not identify significant moderators accounting for the substantial between-study heterogeneity.

### Clinical implications and future directions

The high prevalence of depressive and anxiety disorders among patients with AF undergoing PVI underscores the importance of integrating psychometric diagnostics into standard cardiological practice. A patient-centered approach in diagnosing and treating patients with AF undergoing PVI, based on a bio-psycho-social model, should be considered in future guideline recommendations to support effective management. Additionally, PVI not only effectively manages AF but also significantly improves the associated mental health conditions. The substantial heterogeneity calls for a standardized scientific approach to ensure reliable, evidence-based medical recommendations. By aligning diagnostic tools and treatment protocols, patient outcomes can be optimized and enhance the integration of mental health management into the broader framework of cardiac care.

## Supplementary Information

Supplementary Information 1.

Supplementary Information 2.

## Data Availability

The study-level dataset extracted for the meta-analysis and the analysis scripts used to generate the results are available in the Open Science Framework (OSF) repository at: https://osf.io/nxeh5.
