# Supplementary material for: Depression and anxiety disorders in patients with atrial fibrillation undergoing a pulmonary vein isolation: A systematic literature review and meta-analysis
Source: Sci Rep. 2026 Mar 12;16:8960. doi: 10.1038/s41598-026-42473-4 (PMC12988059; doi:10.1038/s41598-026-42473-4)
Supplement: Supplementary file 1 — Supplementary Information 1. [file 41598_2026_42473_MOESM1_ESM.docx]



Records identified from*:

In Total (n = 2,735)

Databases (n = 2,424)

Pubmed (n = 973)

Web of Science (n = 588)

PsycInfo (n = 13)

Embase (n = 1,152)

Cochrane library (n = 1)

Registers (n = 8 )

Clinical Trials (n = 8)

**Identification**




Records screened

(n = 1,602)

Records excluded**

(n = 1,505)



**Screening**

Reports sought for retrieval

(n = 97)





Reports assessed for eligibility

(n = 97)

Reports excluded:

Reason 1: Congress abstracts (n = 45)

Reason 2: No mental disorder reported (n = 23)

Reason 3: No prevalence rate (n = 14)




Studies included in review

(n = 18)

Reports of included studies

(n = 18)

**Included**

*Consider, if feasible to do so, reporting the number of records identified from each database or register searched (rather than the total number across all databases/registers).

**If automation tools were used, indicate how many records were excluded by a human and how many were excluded by automation tools.

*From:*  Page MJ, McKenzie JE, Bossuyt PM, Boutron I, Hoffmann TC, Mulrow CD, et al. The PRISMA 2020 statement: an updated guideline for reporting systematic reviews. BMJ 2021;372:n71. doi: 10.1136/bmj.n71. For more information, visit: <http://www.prisma-statement.org/>
