# Supplementary material for: Depression and anxiety disorders in patients with atrial fibrillation undergoing a pulmonary vein isolation: A systematic literature review and meta-analysis
Source: Sci Rep. 2026 Mar 12;16:8960. doi: 10.1038/s41598-026-42473-4 (PMC12988059; doi:10.1038/s41598-026-42473-4)

Online supplementary material

Table S1. Search strategy of term for the systematic search in diverse databases

| **Category** | **Terms** |
| --- | --- |
| **Ablation** | Cryoballoon  "Ablation for atrial fibrillation"  "left atrial arrhythmia* ablation"  "left atrial ablation line*”  "ablation for atypical flutter"  "atypical flutter ablation"  "Linear ablation* for atrial fibrillation"  "Ablation outcome* in atrial fibrillation"  "Ablation for Persistent atrial fibrillation"  "Ablation for Paroxysmal atrial fibrillation"  "Pulmonary Vein Isolation"  "Atrial Fibrillation ablation"  "Catheter ablation for atrial fibrillation"  "Circumferential pulmonary vein ablation"  "Radiofrequency ablation for atrial fibrillation"  "Radiofrequency PVI"  "Cryoablation for atrial fibrillation"  "Antral pulmonary vein isolation"  "Segmental pulmonary vein isolation" |
| **Mental disorder** | "mental disorder*"  "mentally ill"  "psychological disorder*"  "psychiatric disorder*"  “psychosomatic disorder*"  "psychiatric illness"  "mental illness"  "psychosomatic illness"  "mental health"  "mood disorder"  "affective disorder*"  Depression  "depressive disorder*"  "major depression"  Anxiety  "anxiety disorder"  "health anxiety"  "post-traumatic stress disorder"  "post traumatic stress disorder"  PTSD  "stress disorder*"  "adjustment disorder*"  "somatoform disorder*"  Somati?ation  "somatic symptom disorder"  "bodily distress disorder"  "atypical pain"  "persistent pain"  "chronic pain"  "somatoform pain"  "irritable bowel syndrome"  "irritable colon"  IBS  "functional disorder*"  "functional somatic symptom*"  "eating disorder*"  "anorexia nervosa"  "bulimia nervosa"  "binge eating disorder" |
| **Quality of life** | "quality of life"  "life quality"  "Well-being"  "well being" |

Table S2. Included studies on patients with depressive and/or anxiety disorders and atrial fibrillation undergoing PVI

| **Year** | **Year** | **Country** | **Sample Size** | **Mean Age** | **Type of atrial fibrillation** | **Proportion of paroxysmal atrial fibrillation (%)** | **Somatic comorbidities**  **(%)** | **Antiarrhythmic Medication** | **JBI** | **Assessment instrument depression disorder** | **Assessment instrument anxiety disorder** | **Main results** |
| --- | --- | --- | --- | --- | --- | --- | --- | --- | --- | --- | --- | --- |
| Al-Kaisey et al. | 2023 | Australia | 52 | 58 | both | 47 | CHF 12  DM 6  Hypertension 53  stroke 8  vascular disease 27  obstructive sleep apnea 14 | Beta-blockers 37  Amiodarone 20  Flecainide 31  Sotalol 39 | 9 | HADS, BDI-II | HADS | Improvement in psychological symptoms of anxiety and depression was observed with catheter ablation,  but not medical therapy |
| Charitakis et al. | 2017 | Schweden | 192 | 60.5 | both | 37 | CHF 9  Stroke 10  DM 8  Hypertension 42  CKD 20 | Beta-blocker 73  Amiodarone 22  Flecainide 18  Dronedarone 12 | 8 | HADS | HADS | Anxiety and depression predicted arrhythmia-related symptoms (e.g. palpitations, breathlessness during activity, tiredness, and worry/anxiety) |
| Du et al. | 2023 | China | 428 | 67 | both | 60.7 | Stroke 6.8  DM 16.8  Hypertension 53  CAD 16.1 | N/A | 8 | HADS | HADS | Depression is common in patients with AF after RF ablation. Intensive management of sedation may be of great importance for reducing symptoms of depression. |
| Efremidis et al. | 2014 | Greece | 57 | 56.9 | paroxysmal | 100 | DM 8.8  Hypertension 47.4  CAD 5.3 | Beta-blocker 21.1  Class I AADs 8.8  Class II AADs 47.4 | 9 | BDI | STAI | There was a significant improvement in anxiety and depression after PVI.  Depression and anxiety were predictors for atrial fibrillation recurrence following PVI |
| Fichtner et al. | 2012 | Germany | 133 | 57 | paroxysmal and persistent | 65.4 | CHF 1.5  DM 6.8  Hypertension 51.1  CAD 10.5 | Beta-blocker 40 | 9 | MDI | WHO-5-  Well-Being-  Index | Significant improvement of depression and anxiety in all patients after 3 months and 4 years. The improvement in depression was significantly greater in patients who underwent a successful ablation than patients with unsuccessful ablation |
| Hasebe et al. | 2020 | Japan | 35 | 60.05 | paroxysmal | 100 | Stroke 6  DM 9  Hypertension 46 | Beta-blocker 34 | 9 | SDS | STAI | Some augmentation of parasympathetic reactivity to stress correlated with reduced anxiety after PVI |
| Hobensack et al. | 2023 | USA | 1293 | 65.5 | paroxysmal | 100 | CHF 44.5  DM 23.9  Hypertension 78.8 | Beta-blockers 75.9  Amiodarone 16.4 | 8 | N/A | clinical | Anxiety is a common symptom in AF patients |
| Jeon et al. | 2017 | Korea | 236 | 55.8 | both | 73.9 | DM 2.5  Hypertension 21.6  CAD 9.75 | N/A | 8 | CESD | STAI-S | Depression and anxiety improved 6 months after PVI. The type of atrial fibrillation significantly affected the timing of recurrence, with persistent atrial fibrillation linked to earlier recurrences |
| Jia et al. | 2013 | China | 335 | N/A | both | 61.2 | CHF 11.04  Stroke 14  DM 18.2  Hypertension 63.6 | N/A | 8 | MHI-5 | N/A | A poorer mental health status is associated with arrhythmia recurrence after catheter ablation |
| Pavlicek et al. | 2022 | Germany | 118 | 64.0 | both | 56 | CHD 11  Stroke 10  DM 17  Hypertension 72  CAD 23  CKD 22 | Beta-blocker 88  Other AAD 43 | 9 | HADS | HADS | Anxiety and depression decreased significantly after PVI in all subgroups regardless of AF recurrence |
| Raileanu et al. | 2023 | Netherlands | 295 | 64.9 | Both | 65.1 | CHD 6.1  Stroke 4.7  DM 7.1  Hypertension 45.4  CAD 14.2  CKD 22 | Beta-blockers 43.7  Amiodarone 14.6  Class I AADs 31.5 | 8 | HADS | HADS | Patients with elevated HADS scores reported a lower quality of life compared to participants with low HADS scores at baseline and 12 months after ablation. However, both groups improved in quality of life, irrespective of their depressive and  anxiety symptoms. |
| Risom et al. | 2019 | Denmark | 276 | 61.1 | both | N/A | CHD 15  DM 7  CAD 18 | N/A | 9 | HADS | HADS | Patients treated for AF or AFL experienced decreased mental and physical health and high readmission rates 6 to 12 months following catheter ablation. |
| Risom et al. | 2023 | Denmark | 929 | 61 | N/A | N/A | CHF 13  DM 8  Hypertension 34  CAD 20 | N/A | 9 | HADS | HADS | High observed readmission rates were associated with risk factors that included anxiety and depression |
| Sang et al. | 2013 | China | 82 | 55.9 | paroxysmal | 100 | CHD 2.4  Stroke 8.5  DM 13.4  Hypertension 64.6  CAD 9.8 | N/A | 8 | SDS | SAS | Catheter ablation is effective in reducing symptoms of depression and anxiety superior to AAD therapy (all P < 0.001) |
| Segan et al. | 2024 | Australia | 338 | 66.1 | persistent | 0 | CHD 47.5  Stroke 5.3  DM 10.4  Hypertension 47  CAD 12.1 | N/A | 8 | HADS | HADS | Females with persistent AF reported worse baseline symptoms and quality of life than males. Despite higher arrhythmia recurrence and more repeat procedures, both sexes had a similarly low AF burden and significant improvements in quality of life and psychological well-being post-ablation. |
| Teppo et al. | 2022 | Finland | 5110 | N/A | N/A | N/A | N/A | N/A | 9 | N/A | N/A | Catheter ablation was used in 2.2 % of patients without mental health conditions, in 2.0% of patients with depression and in 2.8 % of patients with anxiety disorder |
| Walters et al. | 2018 | Australia | 20 | 57 | both | N/A | Stroke 5  DM 10  Hypertension 40 | Beta-blockers 35  Flecainide 10  Sotalol 50 | 9 | HADS | N/A | Effective AF ablation was associated with significant improvements, suggesting AF itself may be a treatable causative factor of distress |
| Yu et al. | 2012 | China | 97 | 55.17 | persistent | 0 | DM 4.08  Hypertension 54.1  CAD 3.06 | Beta-blocker 27.84  Amiodarone 86.6 | 8 | SDS | SAS | Anxiety and depression increase the recurrence risk of persistent AF after PVI. PVI can ameliorate the anxiety and depression symptoms in patients with persistent AF |

*Note.* JBI = Joanna Briggs Institute Critical Appraisal Tools; AF = atrial fibrillation; AFL = atrial flutter; CHD = Congestive Heart disease; DM = Diabetes mellitus; CAD = Coronary artery disease; CKD = Chronic Kidney Disease; AAD = antiarrhythmic drug; RF = radiofrequency; N/A = not available

Figure S1. Funnel plot of studies of depressive disorders

The plot displays the relationship between study effect size and standard error to assess potential publication bias. Each point represents an individual study included in the meta-analysis.


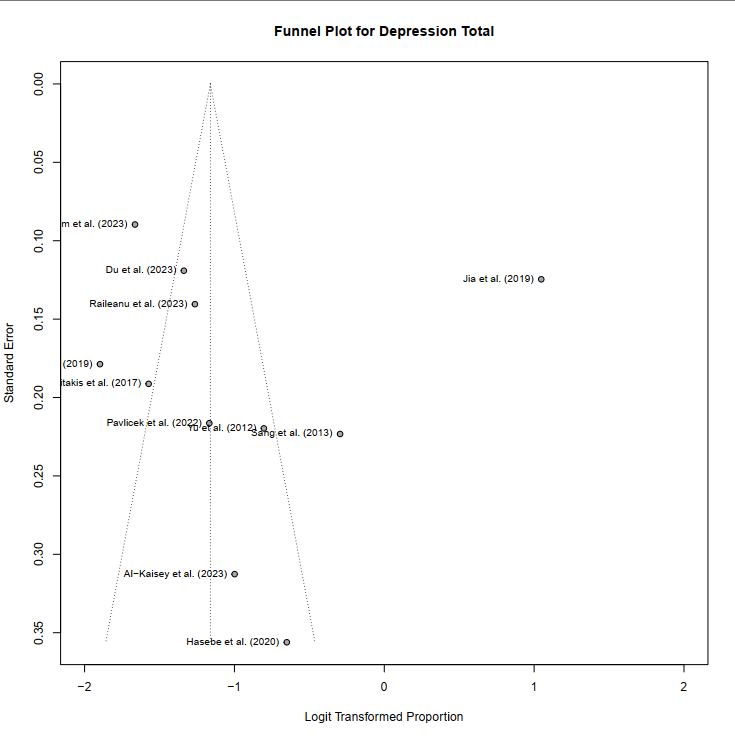


Figure S2. Funnel plot of studies of anxiety disorders

The plot displays the relationship between study effect size and standard error to assess potential publication bias. Each point represents an individual study included in the meta-analysis.


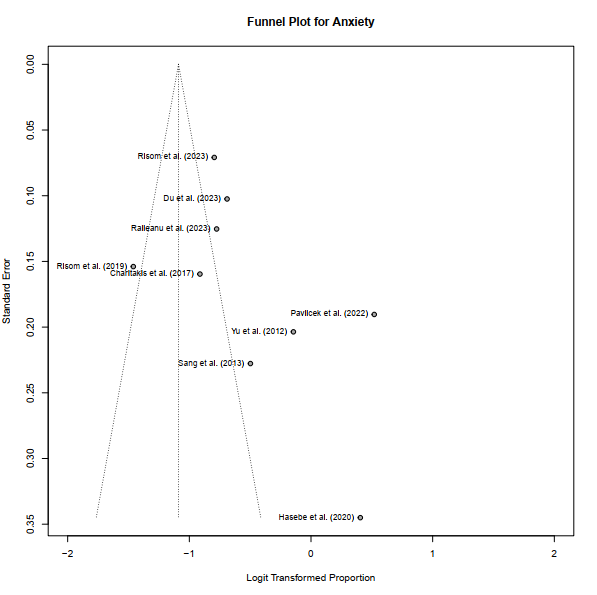

Supplement: Supplementary file 2 — Supplementary Information 2. [file 41598_2026_42473_MOESM2_ESM.docx]
